# Sericin alleviates motor dysfunction by modulating inflammation and TrkB/BDNF signaling pathway in the rotenone-induced Parkinson’s disease model

**DOI:** 10.1186/s40360-023-00703-9

**Published:** 2023-11-07

**Authors:** Zahra Salari, Ghorbangol Ashabi, Ali Fartoosi, Ahmad Fartoosi, Marjan Shariatpanahi, Mehdi Aghsami, Hamed Montazeri, Afshin Kheradmand

**Affiliations:** 1 Department of Pharmacology and Toxicology, School of Pharmacy, Iran University of Medical Sciences, P.O. box: 1475886671, Tehran, Iran; 2 Department of Physiology, School of Medicine, Tehran University of Medical Sciences, Tehran, Iran; 3 Department of Pharmacognosy and Pharmaceutical Biotechnology, School of Pharmacy, Iran University of Medical Sciences, Tehran, Iran

**Keywords:** Sericin, Rotenone, Parkinson’s disease, BDNF, Inflammation, Rat

## Abstract

**Background:**

Parkinson’s disease (PD) is a progressive neurodegenerative disorder characterized by the degeneration of nigrostriatal dopaminergic neurons and movement impairment. Based on theories, neuroinflammatory processes may be vital in the etiology of PD and other neurodegenerative diseases. Reports show that rotenone has neurotoxic, inflammatory, and motor impairment effects in PD. Sericin is a natural polymer with effective properties, such as neuroprotective and anti-inflammatory. Therefore, this study aimed to examine the effects of sericin administration on motor dysfunction by modulating inflammation and tyrosine kinase B/brain-derived neurotrophic factor (TrkB/BDNF) pathway in the rotenone-induced PD model.

**Methods:**

Wistar male rats (3-months-old) were treated with rotenone (2 mg/kg every 48 h for 30 days) to induce a rotenone-induced PD model. Also, sericin was administered orally at dose of 200 mg/kg every 48 h for 30 days. Rotarod and bar tests were performed for motor dysfunction. The protein levels of BDNF, c-fos, TrkB, tumor necrosis factor- α (TNF-α), interleukin-6 (IL-6) and catalase activity were evaluated in the striatum area.

**Results:**

Results showed that sericin increased latent time in the rotarod test and decreased the time staying on the pole in the bar test compared to the PD group (P < 0.001 for both tests). Moreover, sericin treatments decreased TNF-α (P < 0.001) and IL-6 (P < 0.001) concentration levels and enhanced the levels of BDNF (P < 0.001), c-fos (P < 0.001), TrkB (P < 0.001) proteins and catalase activity (P < 0.05) in the striatum area compared to the PD group.

**Conclusion:**

These results support a protective benefit of sericin therapy in a rotenone-induced PD paradigm by reducing motor impairment, inflammatory response, and disruption of the TrkB/BDNF signaling pathway.

**Supplementary Information:**

The online version contains supplementary material available at 10.1186/s40360-023-00703-9.

## Background

Parkinson’s disease (PD) is one of the disorders related to the neurodegeneration of the central nervous system and the second most common disease after Alzheimer’s disease. The incidence of PD is more than 1% of the population over 60 years and 4% of people over 80 years. The main feature of PD is the death of dopaminergic neurons located in the nigrostriatal pathway in the midbrain [1]. The loss of dopamine-producing neurons causes motor symptoms of PD, including tremors, muscle stiffness, slowness, and difficulty moving and stepping. In addition, in the later stages of the disease and with its progression, cognitive, behavioral, and dementia problems may occur [2, 3]. Based on studies, ample evidence suggests a link between inflammation, oxidative stress, and PD in the substantia nigra [4], [5]. Although the main cause of this disease has not been discovered yet, scientists have identified factors such as environmental toxins, genetic history, oxidative stress, specific viruses, or the outcome of all these cases as the main cause of PD [5]. Notably, only 15% of instances of PD have genetic factors. Researchers consider environmental factors and, most importantly, toxins used in insecticides and herbicides to be important factors in the incidence of PD [6], [7]. Environmental toxins, such as rotenone, cause movement problems and clinical characteristics in rats comparable to those caused by PD. These traits include the selective degradation of the nigrostriatal dopaminergic pathway [8]. Rotenone, as a pesticide, plays a significant role in causing mitochondrial dysfunction in PD [9]. Rotenone involves in numerous pathogenic pathways that mediate dopaminergic cell death, including oxidative stress, α-synuclein phosphorylation and aggregation, Lewy bodies pathology, DJ-1 acidification and translocation, proteasomal dysfunction, and nigral iron accumulation [10].

Sericin and fibroin are the two main peptides that compose the protein polymers known as silks [11]. Antioxidant, anti-tumor, anti-inflammatory, antimicrobial and antibacterial, moisturizing, wound-healing, and anti-tyrosinase activities are just a few of its biological qualities [12], [13]. Research has demonstrated that sericin, via modifying the protein kinase B (AKT) signal transduction pathway, possesses anti-apoptotic characteristics in the hippocampus region of diabetic rats [14]. Researchers have found that sericin stops lipid peroxidation, gets rid of reactive oxygen species (ROS), and boosts the antioxidant functions of enzymes in the brain, including superoxide dismutase (SOD), catalase (CAT), glutathione reductase, and glutathione (GSH) [15]. In addition, several experimental studies have shown that sericin mainly protects dopaminergic and cholinergic neurons through its antioxidant effects [12].

Brain-derived neurotrophic factor (BDNF) is involved in the pathophysiology of PD and L-DOPA-induced dyskinesias. These effects have been attributed to changes in BDNF levels in the substantia nigra and cerebral cortex [13], [14], [15]. Binding of BDNF to the tropomyosin-related kinase receptor type B (TrkB), occurs in several regions of the human brain, indicating its widespread distribution. The production and activation of BDNF and TrkB primarily occur within the dopaminergic neurons located in the substantia nigra. This signaling pathway plays a crucial role in various neurophysiological processes, such as neuroprotection, neuronal maturation, and the maintenance of neuronal integrity [16]. BDNF increases cAMP-response element binding protein (CREB) activation, and CREB can be phosphorylated by several protein kinases, making it a convergent target for many intracellular signaling pathways, such as immediate early genes (IEGs) like the inducible transcription factor c-fos [17]. The c-fos biochemically activates neurons in the basal ganglia in the basal ganglia in response to dopamine-mimetic compounds in PD in an experimental model [18].

According to shreds of evidence, cytokines such as interleukin-1 β (IL-1β), interleukin-6 (IL-6), and tumor necrosis factor-α (TNF-α), are thought to play a key role in the occurrence and progression of PD, and measuring their levels in vivo is essential for making an early diagnosis of the disease. TNF-α is a potent cytokine with cytotoxic and stimulatory effects that play a crucial role in immune system signaling. According to research, Parkinson’s patients had significantly higher quantities of TNF-α, a glial cell-related cytokine, in their brains and cerebrospinal fluid. Additionally, there is a robust negative relationship between IL-6 levels and PD severity [19], [20].

Based on these findings, we investigated sericin’s effect on motor function by measuring latent time in rotarod and bar tests in the rotenone-induced PD animal model. Moreover, we aimed to assess the protein levels of TrkB, BDNF, c-fos, the concentration of TNF-α and IL-6 and catalase activity in the striatum of the brain of rotenone-injected rats.

## Materials and methods

### Animals

This research used male Wistar rats aged 3 months and weighing 280–300 g, purchased from the Pasteur Institute of Iran. Rats were kept in polycarbonate cages under controlled conditions with 12-hour light-dark cycles, a temperature of 23 ± 2 °C, and a humidity of 60 ± 5%. Standard concentrate food and sufficient water were available to the animals during the entire maintenance period, except for the experimental period. All experiments were performed according to the guidelines for using laboratory animals by ARRIVE guidelines (2019) and the US National Institutes of Health, as approved by the Iran University of Medical Sciences Ethics Committee with approval number IR.IUMS.REC.1398.353.

### Experimental groups

**Fig. 1:**
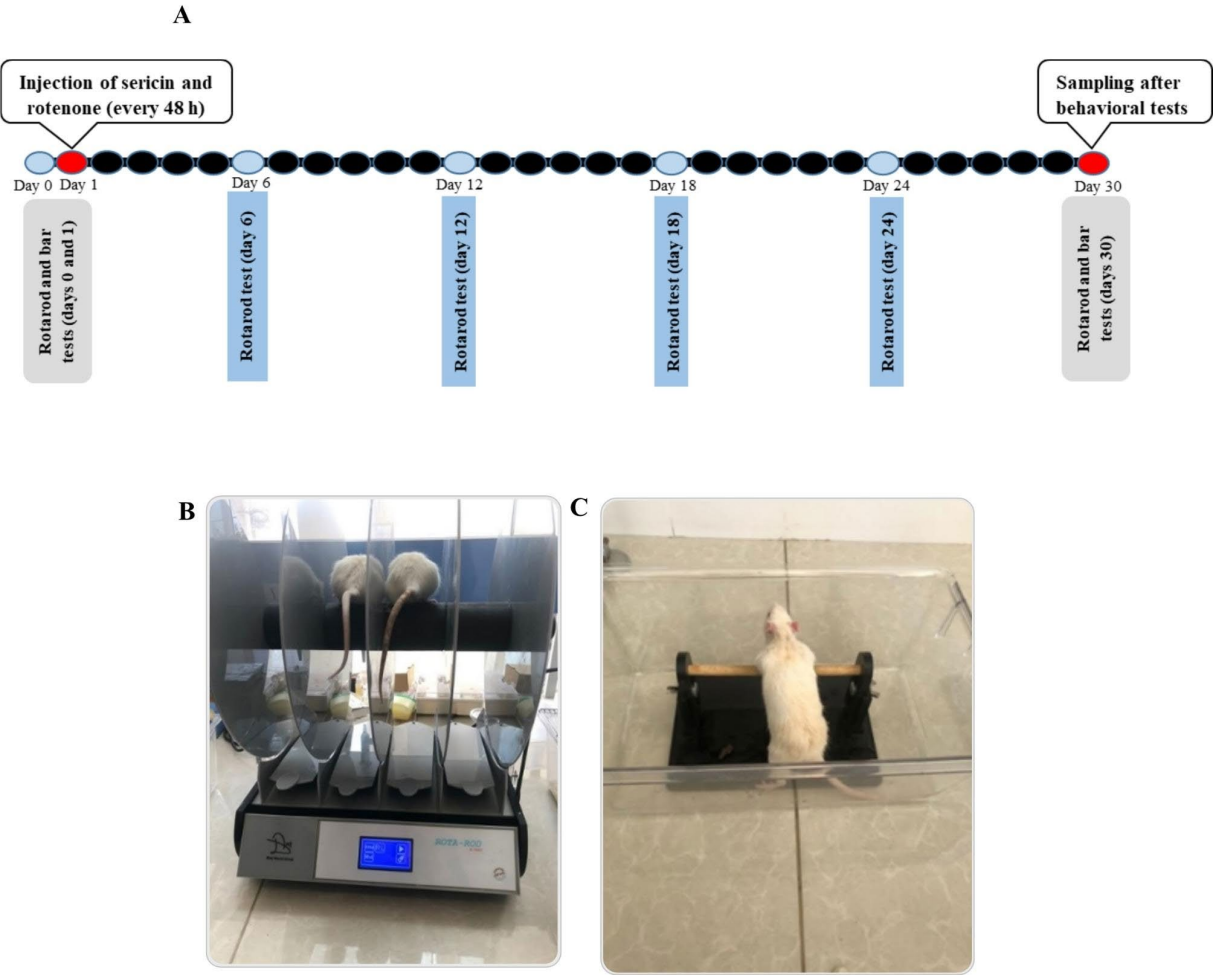
(A) The time line of the experiment. (B) rotarod test. (C) bar test

Rats are randomly divided into five groups. (1) The control group which received normal food and water only (control group). (2) Rotenone vehicle group that received 5% DMSO subcutaneously every 48 h for 30 days (DMSO group). (3) Rotenone was administered subcutaneously at the dose of 2 mg/kg every 48 h for 30 days (rotenone (or PD) group). (4) Sericin (200 mg/kg) was given orally every 48 h for 30 days (sericin group). (5) Rotenone was administered subcutaneously at the dose of 2 mg/kg every 48 h for 30 days, and sericin (200 mg/kg) was given orally every 48 h for 30 days (rotenone (PD) + sericin group) (Fig. 1A). All behavioral and molecular experiments were done by an operator without knowledge of the experimental conditions.

### Drugs preparation

Rotenone (R8875-5G, purchased from Sigma-Aldrich) was prepared in DMSO and sunflower oil solvents for injection at 2 mg/kg. Groups 3 (rotenone (PD) group) and 5 (rotenone (PD) + sericin group) received rotenone as a PD model. It should be noted that DMSO solvent was used in a non-toxic concentration of 5 to 10% and only for the preparation of rotenone stock solution, and then it was brought up to the final volume with sunflower oil. For sericin solution preparation, (Sigma-Aldrich) 200 mg/kg of sericin was made by dissolving it in distilled water [21–24]. All solutions were used freshly.

### Behavioral-motor tests

#### Rotarod test

The rotarod test was conducted at baseline (before the first day of rotenone injection), 1, 6, 12, 18, 24, and 30 days during treatment [25], [26]. On the first day, the rotation speed was 11 rpm, and by the end of the training, it had increased to 15 rpm [27], [28] (Fig. 1B).

#### Bar test

Rats were positioned on a bar with their forepaws in the posture of half-rearing, 10 cm above the base, in order to measure their stiffness and catalepsy. The time of the bar test was then recorded as the moment one or both paws were removed. The cutoff was thought to be 180 s of delay [29] (Fig. 1C).

#### Striatal tissue extraction

On day 30, rats were anesthetized intraperitoneally by ketamine (70 mg/kg) and xylazine (10 mg/kg) injection. During anesthesia, animals were euthanized with a higher dose of ketamine (450 mg/kg) and xylazine (30 mg/kg). Then, the brain was removed, and the striatum area of the brain was extracted according to the coordinates of Paxinos and Watson [30] and kept at -80 °C until use.

#### ELISA detection kit

Fifty mg of striatal tissue samples were homogenized in buffer lysis (150 mM NaCl, 0.5% sodium deoxycholate, 0.1% sodium dodecyl sulphate, 50 mM Tris-HCl pH 7.5, cocktail protease inhibitor) for 20 min at 4 °C (n = 6). Lysed samples were centrifuged at 15,000 rpm for 5 min at 4 °C. The supernatant was taken out, the concentration of proteins was assessed using Bradford methods [31], and samples were tested following the guidelines (Cusabio Co. [Cat no. CSB-E11987r and Cat no. CSB-E04640r]). Using this method, IL-6 and TNF-α were subjectively assessed in striatal tissue samples. Using a microplate reader (BioTek Instrument) at a wavelength of 450/570 nm, the optical density of each well was calculated.

#### Western blotting

The frozen striatal tissue samples were homogenized for 20 min at 4 °C in buffer lysis (150 mM NaCl, 0.5% sodium deoxycholate, 0.1% sodium dodecyl sulphate, 50 mM Tris-HCl pH 7.5, cocktail protease inhibitor), and then the samples were centrifuged at 15,000 g for 5 min at 4 °C to extract the total protein from the supernatant. Total protein concentration was measured by the Bradford method [31]. A 12% polyacrylamide gel was used to separate equal amounts of protein using sodium dodecyl sulfate-polyacrylamide gel electrophoresis (SDS-PAGE). Each gel’s lanes were augmented with pre-stained protein markers to guarantee that electrophoretic transfer was successful. Following an electrophoretic transfer to a polyvinylidene fluoride membrane (Thermo Fisher Scientific), the blots were incubated in a blocking buffer (5% nonfat dry milk in Tris-buffered saline containing 0.05% Tween-20, TBS-T) for an hour at room temperature. The primary antibodies (1/1000, BDNF, TrkB, and c-fos) were then incubated overnight at 4 °C. The blots were treated with the chemiluminescent HRP substrate (Merck Millipore), washed three times for ten seconds in TBS-T, then incubated with the appropriate secondary antibody (1/3000, cell signaling) for 1.5 h at room temperature (Kodak). Following a 15-minute incubation period at room temperature with stripping buffer, the blots were exposed by being re-blocked for 3 h at room temperature with the corresponding β-actin antibody (1/1000, cell signaling), washed for 3–10 min in TBS-T, and then incubated for 2 h with the corresponding secondary antibody (1/2000, cell signaling, USA). Finally, antigen imaging and radiography film exposure was done. After removing the background (n = 6), the cumulative density of the bands was calculated using Image J to examine the data. The densitometric scan of the films revealed data.

#### Catalase activity assay

The aim of the assay is to determine the catalase activity of the samples. Catalase was inhibited by a flux of O^2−^ generated in situ by the aerobic oxidase reaction. H_2_O_2_ started the reaction. The measurement of catalase activity was based on the disposable rate of H_2_O_2_, which was measured at 240 nm by a spectrophotometer by the method of Aebi [32] with some modifications [33]. Thirty µg ml of protein sample lysate was added to a cuvette containing 1.995 ml of phosphate Buffer (50 mM Potassium Phosphate Buffer, pH 7.0 at 25 °C). One ml of freshly prepared H_2_O_2_ (30 mM) was added to the samples and, then read by a spectrophotometer (240 nm).

### Data analysis

In this study, a two-way ANOVA and Tukey’s post hoc test were utilized to examine the behavioral data from the rotarod test (n = 8). A one-way ANOVA followed by Tukey’s post hoc test was used for the bar test (n = 8), Western blot and ELISA data (n = 6) and catalase activity (n = 5). DMSO group that was n = 3 in Western blot and ELISA data analysis. The SPSS ver.18 software is used to analyze all of the data. P < 0.05 was considered a significant level. Values are defined as mean + S.E.M.

## Results

### Sericin’s impact on movement tests in the rotenone-induced PD model

**Fig. 2:**
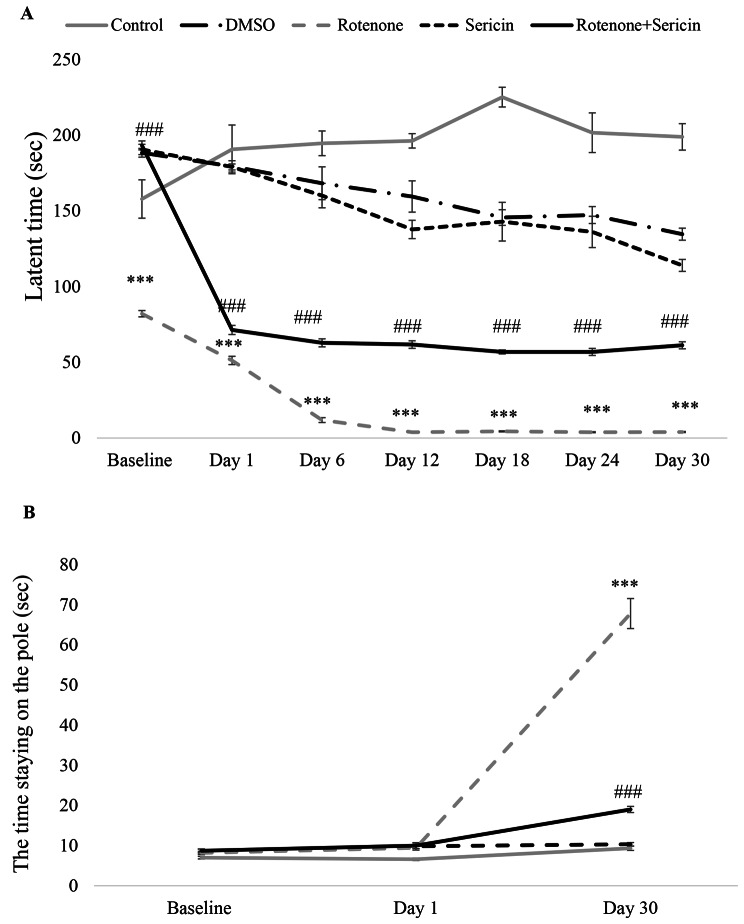
(A) Motor performance of the experimental groups in the rotarod. Spent time in the rotarod task was measured as “latent time”. (B) Performance of experimental groups in the bar test. Amount of time it takes for the rat to release one or both hands from the bar was measured as “time staying on the pole”. Values are defined as mean + S.E.M. ***P < 0.001 indicates a significant difference between the control group and other experimental groups on the same day. ###P < 0.001 indicates a significant difference between rotenone + sericin group compared to rotenone group on the same day

Based on the results, in the rotenone-received animals, balance performance and muscle strength gradually decreased during the experimental days, and on the 30th day, these symptoms significantly decreased compared to the first day [F (4,26) = 13.56, P < 0.001]. In addition, the statistical analysis of the data indicated a significant reduction in motor function between the rotenone-receiving group and the control group (P < 0.001). Data showed an improvement in movement in the rotenone + sericin group compared to the rotenone group (P < 0.001) (Fig. 2A).

The bar test was performed to measure muscle stiffness in the rat. This test was performed in all experimental groups after the rotarod test on days 0, 1, and 30 [F (4, 26) = 1.06); P < 0.001]. The results obtained from this test showed that the amount of catalepsy and muscle stiffness increased significantly in the rotenone-received group compared to the control group (P < 0.001). Also, catalepsy and muscle stiffness were significantly reduced in the rotenone + sericin group compared to the rotenone group. While in other groups, there was no significant difference between the first day and the 30th day (P > 0.05) (Fig. 2B).

### The effect of sericin on the expression of TrkB, BDNF, and c-fos proteins in the rotenone-induced rat model

**Fig. 3:**
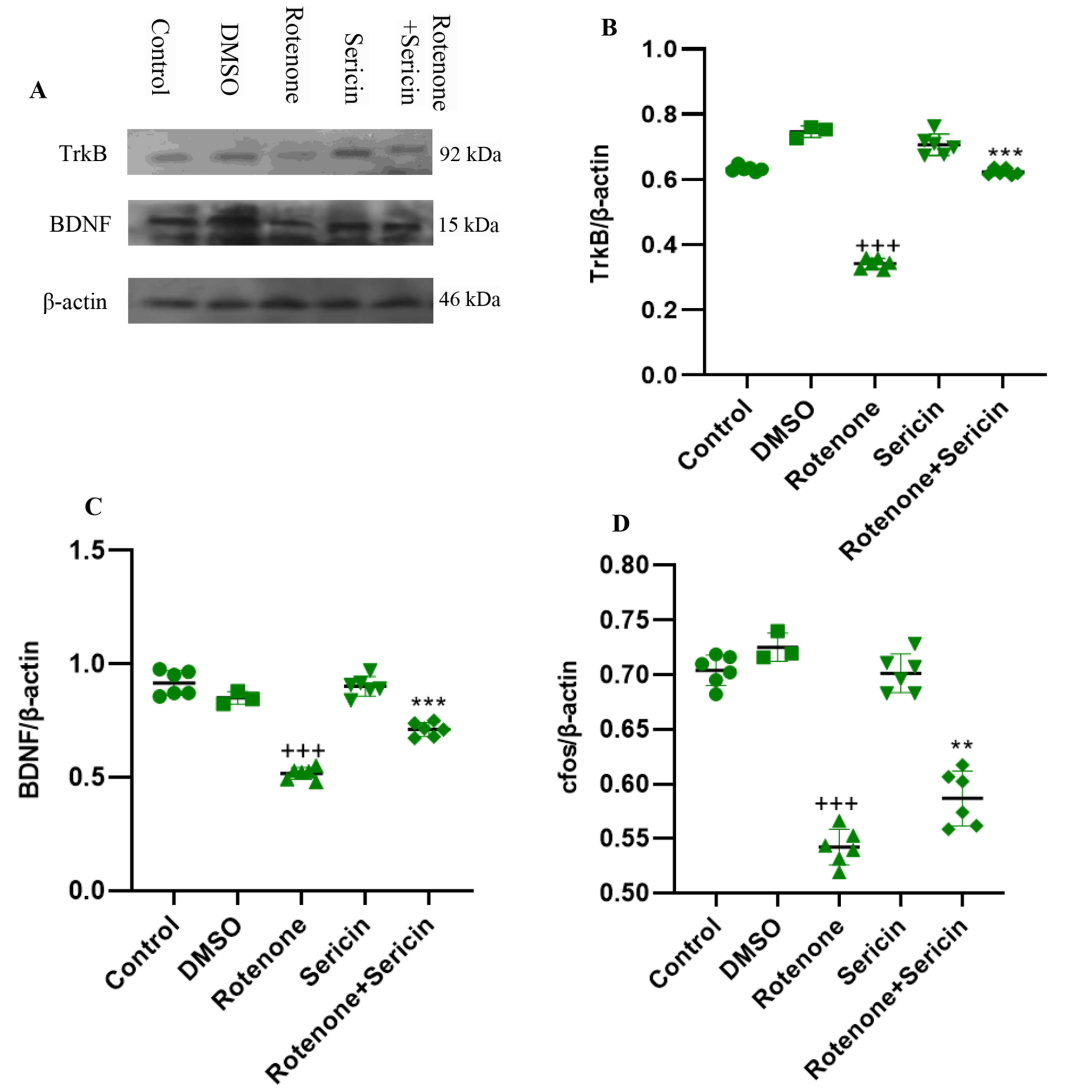
(A) The protein blot of BDNF, c-fos and TrkB in the brain striatum tissue in experimental groups. (B) Densitometric analysis of TrkB to β-actin ratio. (C) Densitometric analysis of BDNF to β-actin ratio. (D) Densitometric analysis of c-fos to β-actin ratio. Values are defined as mean + S.E.M. +++P < 0.001 versus control, **P < 0.01, ***P < 0.001 versus rotenone

This work aimed to examine the impact of sericin on the expression of TrkB, BDNF, and c-fos proteins using the Western blotting technique (Fig. 3A). Densitometric analysis showed that there were significant changes among the groups in the striatal level of TrkB [F (4, 26) = 367.55, P < 0.001], BDNF [F (4, 26) = 106.01, P < 0.001], and c-fos [F (4, 26) = 103.28, P < 0.001] proteins.

The findings of the study indicated a significant reduction in the level of TrkB protein in the rotenone group as compared to the control group (P < 0.001). Additionally, the experimental group treated with both rotenone and sericin exhibited a statistically significant increase in comparison to the group treated with rotenone alone (P < 0.001, as depicted in Fig. 3B). The protein level of BDNF in the rotenone group has significantly decreased compared to the control (P < 0.001). Examination of BDNF protein in different groups showed a significant increase in the level of BDNF in the rotenone + sericin group compared to the rotenone group (P < 0.001, Fig. 3C).

The level of c-fos protein was also investigated, and it was found that rotenone significantly decreased the c-fos level in the striatum compared to the control group (P < 0.001). While the rotenone + sericin group had a significant increase in the c-fos level compared to the rotenone group (P < 0.001, Fig. 3D).

### The effect of sericin on the levels of IL-6 and TNF-α in a rotenone-induced PD model

**Fig. 4:**
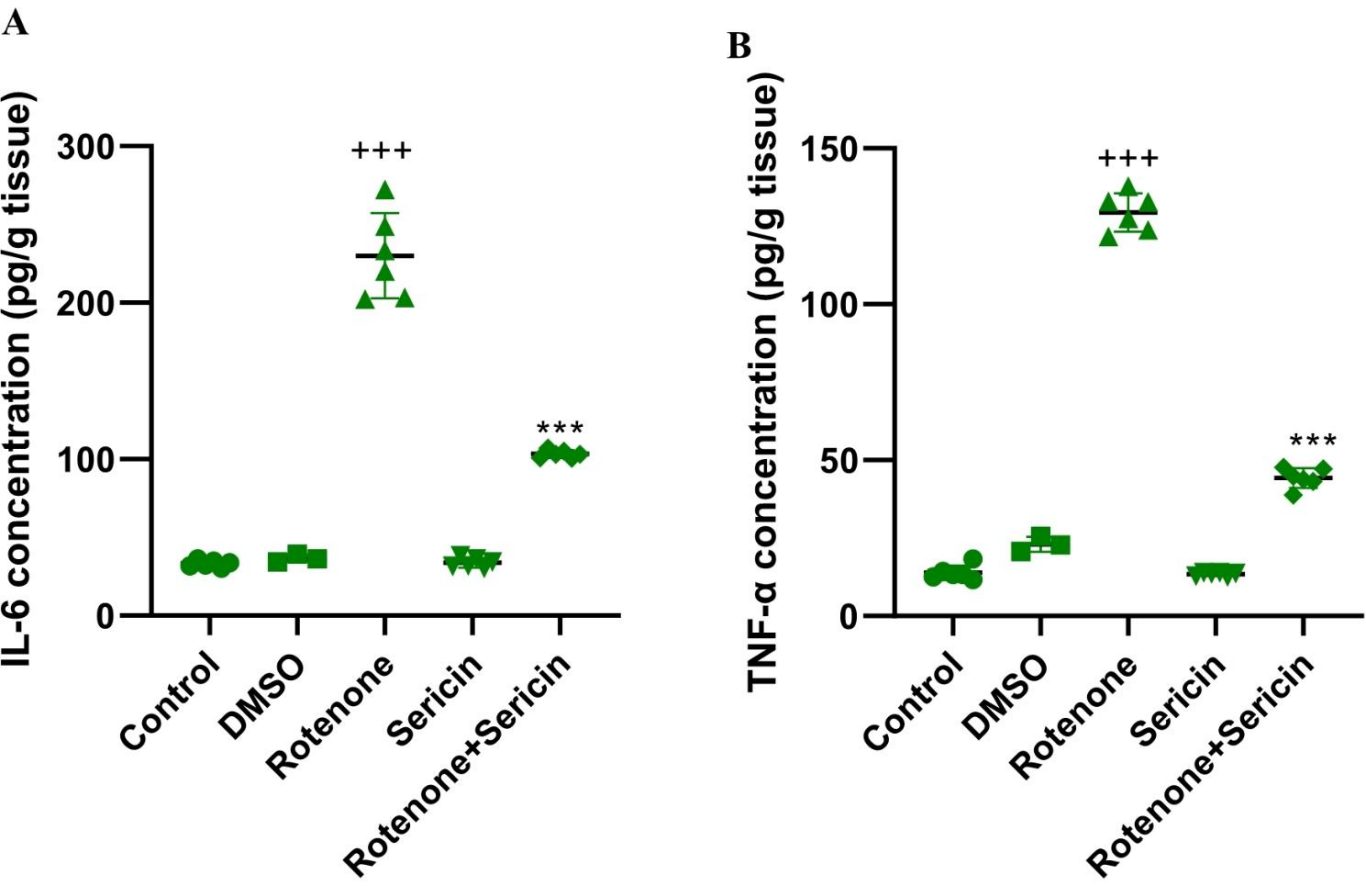
(A) The level of IL-6 was measured by ELISA enzyme detection method. (B) The level of TNF-α was measured by ELISA enzyme detection method. Values are defined as mean + S.E.M. +++P < 0.001 versus control, ***P < 0.001 versus rotenone

In this research, the performance of the ELISA method for detecting inflammatory factors such as concentrations of IL-6 [F (4, 26) = 236.22, P < 0.001] and TNF-α [F (4, 26) = 1097.61, P < 0.001] was investigated among experimental groups. The findings of the study revealed a statistically significant elevation in the IL-6 level within the rotenone group as compared to the control group (P < 0.001). Furthermore, the application of sericin in combination with rotenone resulted in a significant reduction in IL-6 levels compared to the rotenone group (P < 0.001, as shown in Fig. 4A). Assessment of TNF-α illustrated that rotenone increased TNF-α levels compared to the control group (P < 0.001). Treatment of rotenone-induced rats with sericin decreased TNF-α striatal levels compared to the rotenone group (P < 0.001, Fig. 4B).

### The effect of sericin on the catalase activity in the striatum of a PD animal model

Table 1 presented catalase activity in the striatum of a rotenone-induced model in the presence of sericin. Data showed that rotenone decreased catalase activity in comparison to the control group (P < 0.001). Also, data revealed that treatment of rotenone-received rats with sericin increased catalase activity in comparison to the rotenone group (P < 0.05). Although there is a significant difference between the control group and the rotenone + sericin group (p < 0.05).

## Discussion

The present study’s findings present that rotenone injection results in motor impairments, which indicates that the PD model can be induced by rotenone administration. It has been established that rotenone injection generated progressive mobility dysfunction and reduced the death rate [34], [27]. In the current study, rotenone administration has no statistically significant effect on animal weights among experimental groups. The results of the rotarod and bar tests confirm that motor dysfunction occurs in rotenone-injected rats. Following reports, the rotenone-induced animal model may show neuropathological symptoms of PD as well as motor and non-motor behavioral patterns [35]. This PD model has shown that motor impairment symptoms, such as catalepsy and impaired locomotion, appeared in rotenone-treated animals [35], [34]. It is well known that decreased human dopamine activity results in PD symptoms such as rigidity and akinesia. Alam and collagenous disclosed that rotenone increased cataleptic behavior in the animal model, even in low and medium doses [36]. Wrangel et al. (2015) reported that time spent in the rotarod test reduced while animals received rotenone [37].

Data find out that sericin administration prevents movement failure by the rotenone in the rotarod and bar tests. Findings are in agreement with Kim and colleagues, who evaluated silk amino acid’s effects on physical function in PD model rats. It was earlier established by Mohammadi et al. 2019 that sericin treatment at dose of 200 mg/kg for a month reduced anxiety- and depressive-like behaviors [21, 24]. Previous studies [22–24] revealed that 200 mg/kg of sericin for two or three weeks could improve behavioral outcomes in motor function and memory. Based on these studies, in preliminary exploration of current study, 200 mg/kg of sericin was administrated orally for one month every two days to establish that this dose is not toxic.

Our study reveals that PD rats has reduced BDNF, c-fos, and TrkB protein levels in the striatum. BDNF, as a key neurotrophic factor, regulates how neurons in the central and peripheral nervous systems survive, develop and differentiate [38]. Due to its neuroprotective properties, BDNF has been associated with neurodegenerative diseases, including PD. [39]. Lack of neurotrophins, particularly BDNF, may contribute to the pathophysiology of PD as the nigrostriatal dopaminergic neurons continue to degenerate [40]. Li and collaborators described that reduction in BDNF mRNA caused striatal developmental abnormalities, such as morphological damage and motor impairments [41]. BDNF treatment positively affected neurodegeneration and motor dysfunction in animal models [42]. In parallel with our data, some studies have disclosed that BDNF protein expression is decreased in the substantia nigra of PD animals [43]. It has been hypothesized that decreased nigral BDNF resulted in a lack of trophic support, contributed to the loss of nigral dopaminergic neurons in PD model [44]. Jin et al.‘s research in 2020 suggested that PD patients had dramatically decreased BDNF/TrkB levels in the substantia nigra [16]. In our study, sericin effectively increases BDNF, c-fos, and TrkB expression in the substantia nigra in the rotenone-injected rat model. Previous studies have shown that the silk proteins can significantly improve brain function and protect against neurodegenerative disorders [45]. Vatandoust and co-workers reported that sericin reduced cognitive impairment and enhanced BDNF expression in mice with learned helplessness disorder [46]. Studies suggested that c-fos is crucial for regulating neuronal cell survival. C-fos modulates the expression of BDNF both in vivo and in vitro. It has been established that c-fos is a genetic regulator of cellular processes regulating neuronal survival in the brain. In current data, the striatal level of c-fos is increased during sericin treatment [18].

Apoptotic cell death could result from PD’s elevated cytokine levels and decreased neurotrophins levels [47]. Studies on cytokines and neurotrophins in the postmortem brain of experimental PD animals with 1-methyl-4-phenyl-1,2,3,6-tetrahydropyridine or 6-hydroxy dopamine found higher levels of pro-inflammatory cytokines like TNF-α as well as decreased levels of neurotrophins like BDNF in the nigrostriatal regions [40]. The expression of BDNF is prevented by inflammatory factors [48]. Furthermore, a study proposed that reduction in BDNF expression caused increase in IL-1 and TNF-α levels in neuronal cells [49]. The mechanism of inflammatory factors action is as follows: NF-κB binds to DNA and prevents the expression of BDNF; the activity of NF-κB is inhibited by decreasing inflammatory factor and TNF-α levels [50], [51]. Moreover, in a clinical study, the measurement of growth factors and interleukins, especially IL-6, showed significant changes in Parkinson’s patients [52].

It is widely acknowledged that oxidative stress can cause inflammatory responses by producing TNF-α and causing NF-κB inhibitors to degrade [53]. Sericin may have protective effects against stress-induced oxidative stress by restoring mitochondrial activity. Multiple lines of evidence indicating that sericin possess anti-oxidative effect in the in vitro and in vivo contexts [54], [55], [56, 57]. Similarly, Kumar and co-workers demonstrated that silk sericin protected mouse fibroblast cell lines from oxidative stress by suppressing lipid peroxidation and scavenging ROS [58]. Based on our data, sericin could decrease the levels of IL-6 and TNF-α in the rotenone-induced group.

The capacity of sericin to exhibit this ability is ascribed to its ability to hinder the synthesis of pro-inflammatory cytokines such as TNF-α, IL-1, and IFN-γ, in addition to its ability to suppress the COX-2 enzyme and the generation of nitric oxide [61], [62]. Aramwit et al. (2009) conducted an in vivo investigation demonstrating that the administration of sericin for seven days enhanced wound healing in rat back tissue. This effect was attributed to the downregulation of TNF-α and IL-1 production [62]. Additionally, aberrant IL-6 circulation levels in PD patients’ blood are strongly correlated with physical and cognitive aging parameters [59]. The three cytokines, IL-1b, IL-6, and TNF-α, had altered physiological levels in PD [60, 61]. In parallel with activation of inflammatory factors, catalase activity decreases in rotenone group and sericin administration enhances catalase activity, which establishes the antioxidative ability of sericin. This hypothesis is supported by several lines of evidence in different organs, such as ovary and liver [62, 63]. The aforementioned compelling data suggested that three cytokines -IL-1β, IL-6, and TNF-α- play significant role in the occurrence and progression of PD.

Current investigation shows that chronic sericin administration decreases neurodegeneration and improves movement disturbance in the rotenone-induced PD model. Moreover, sericin treatment might increase neuronal plasticity through TrkB/BDNF pathways and diminish neuroinflammation. The current study focuses on some inflammatory factors and TrkB/BDNF pathways. These proteins were only assessed by Western blotting and ELISA methods; it was better to confirm these proteins by immunohistochemistry method. Moreover, rotenone could affect the dopaminergic system in the brain. Finding the importance of sericin on dopamine receptors might be another limitation of this study; authors are planning to evaluate these receptors in future research.

**Table 1:** Effect of sericin on catalase activity in striatum against rotenone-induced toxicity (n = 5). Values are defined as mean + S.E.M. ***P < 0.001, *P < 0.05 versus control, +P < 0.05 versus rotenone

Groups	Control	DMSO	Rotenone	Sericin	Rotenone + Sericin
**Catalase activity (U /mg protein)**	9.57 ± 0.29	10.86 ± 0.56	6.86 ± 0.13***	8.71 ± 0.21	8.31 ± 0.38*+

### Electronic supplementary material

Below is the link to the electronic supplementary material.

Supplementary Material 1

Supplementary Material 2

Supplementary Material 3

## Data Availability

The data sets used and analyzed during the current study are available in the submitted files.

## Declarations

The present study was performed at the Iran University of Medical Sciences. All the animal experiment procedures performed in this research were approved by the Ethics Committee of the Iran University of Medical Sciences, Tehran, Iran, which is under international guidelines for animal experiments. (Approval code: IR.IUMS.REC.1398.353).
