# Supplementary material for: Sericin alleviates motor dysfunction by modulating inflammation and TrkB/BDNF signaling pathway in the rotenone-induced Parkinson’s disease model
Source: BMC Pharmacol Toxicol. 2023 Nov 7;24:60. doi: 10.1186/s40360-023-00703-9 (PMC10631121; doi:10.1186/s40360-023-00703-9)
Supplement: Supplementary file 1 — Supplementary Material 1 [file 40360_2023_703_MOESM1_ESM.pptx]

## Slide 1
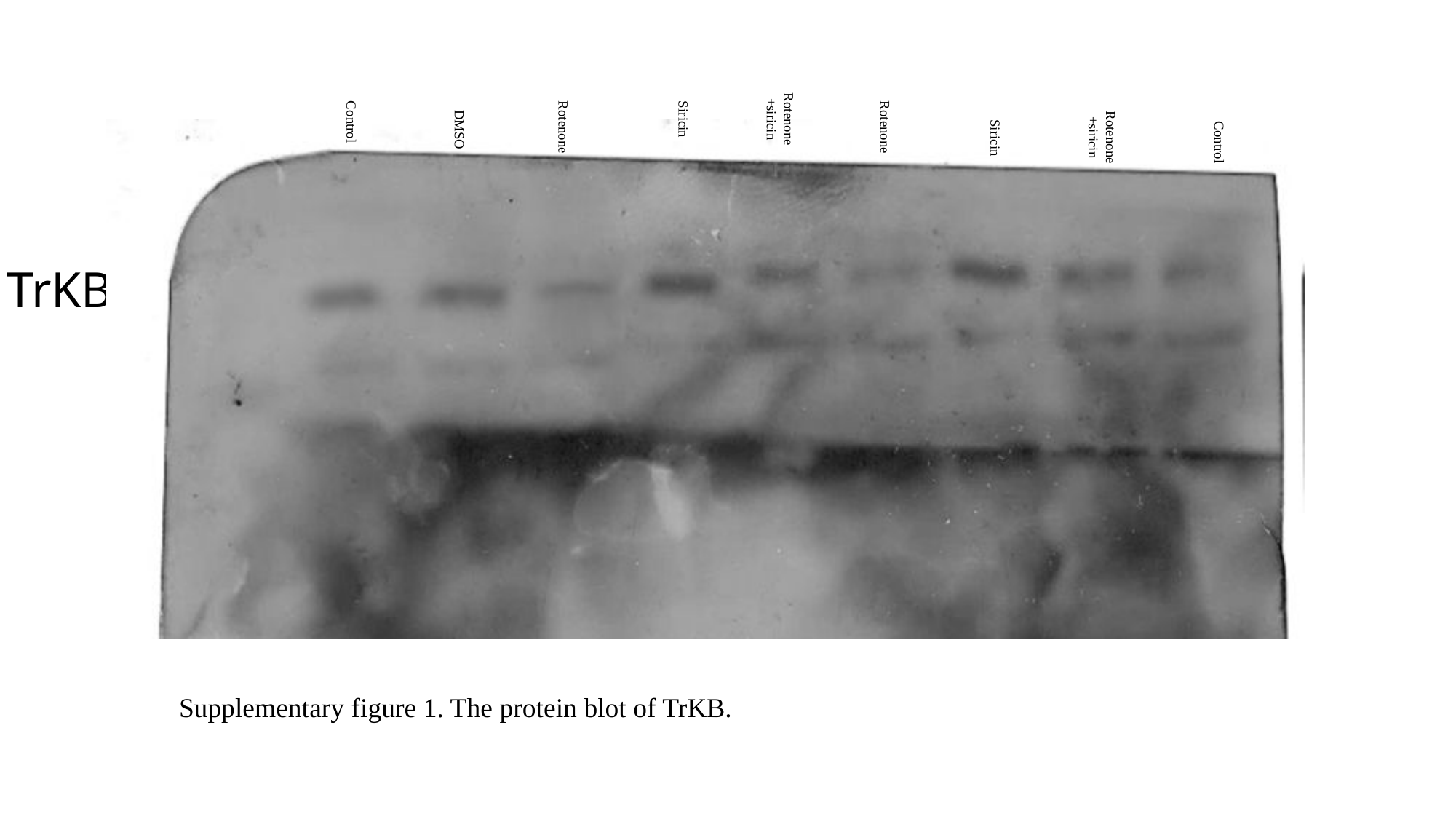

Rotenone+siricin
Control
Rotenone
Siricin
Rotenone
Rotenone+siricin
DMSO
Siricin
Control
# TrKB
Supplementary figure 1. The protein blot of TrKB.

## Slide 2
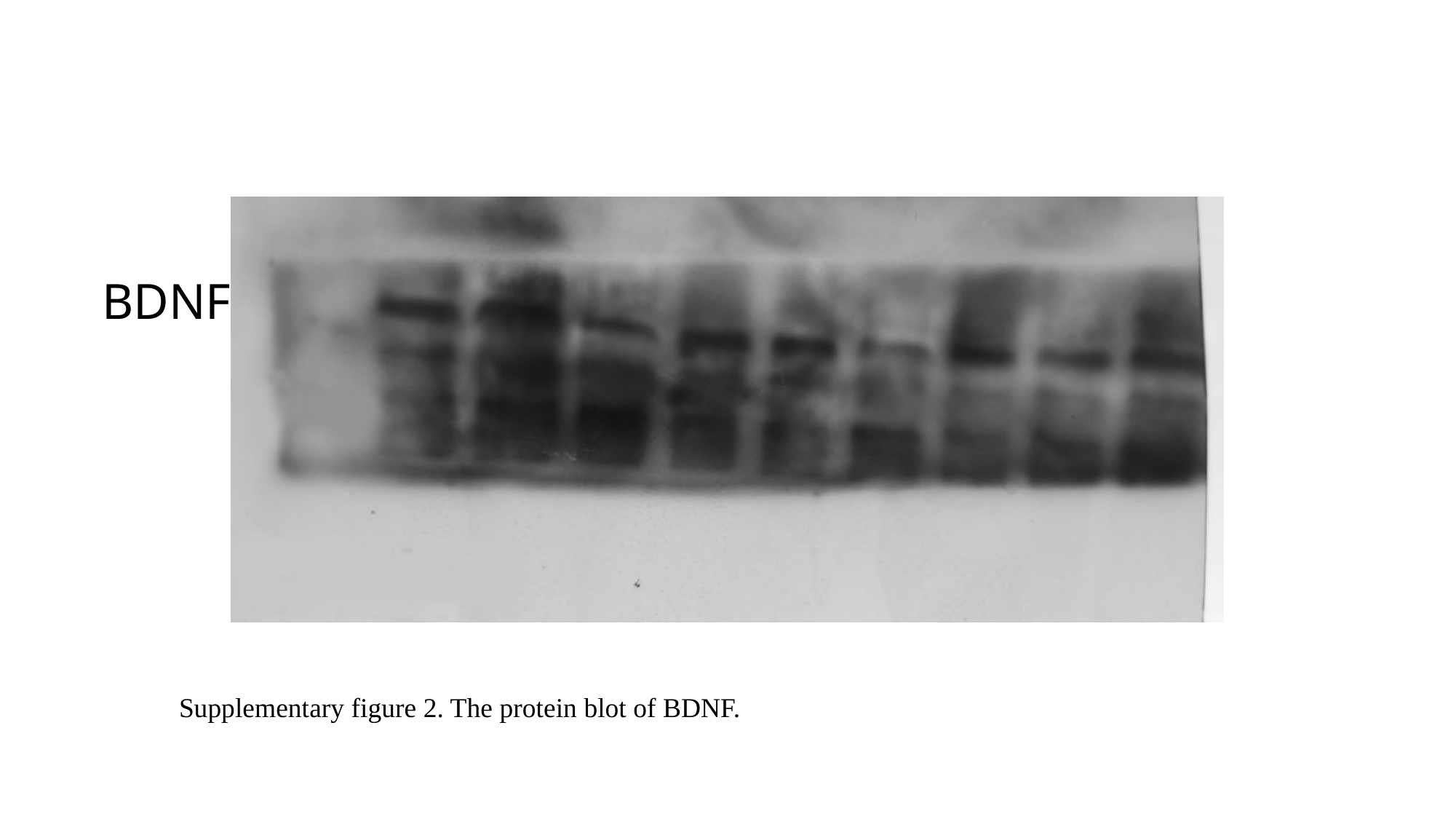

#
BDNF
Supplementary figure 2. The protein blot of BDNF.

## Slide 3
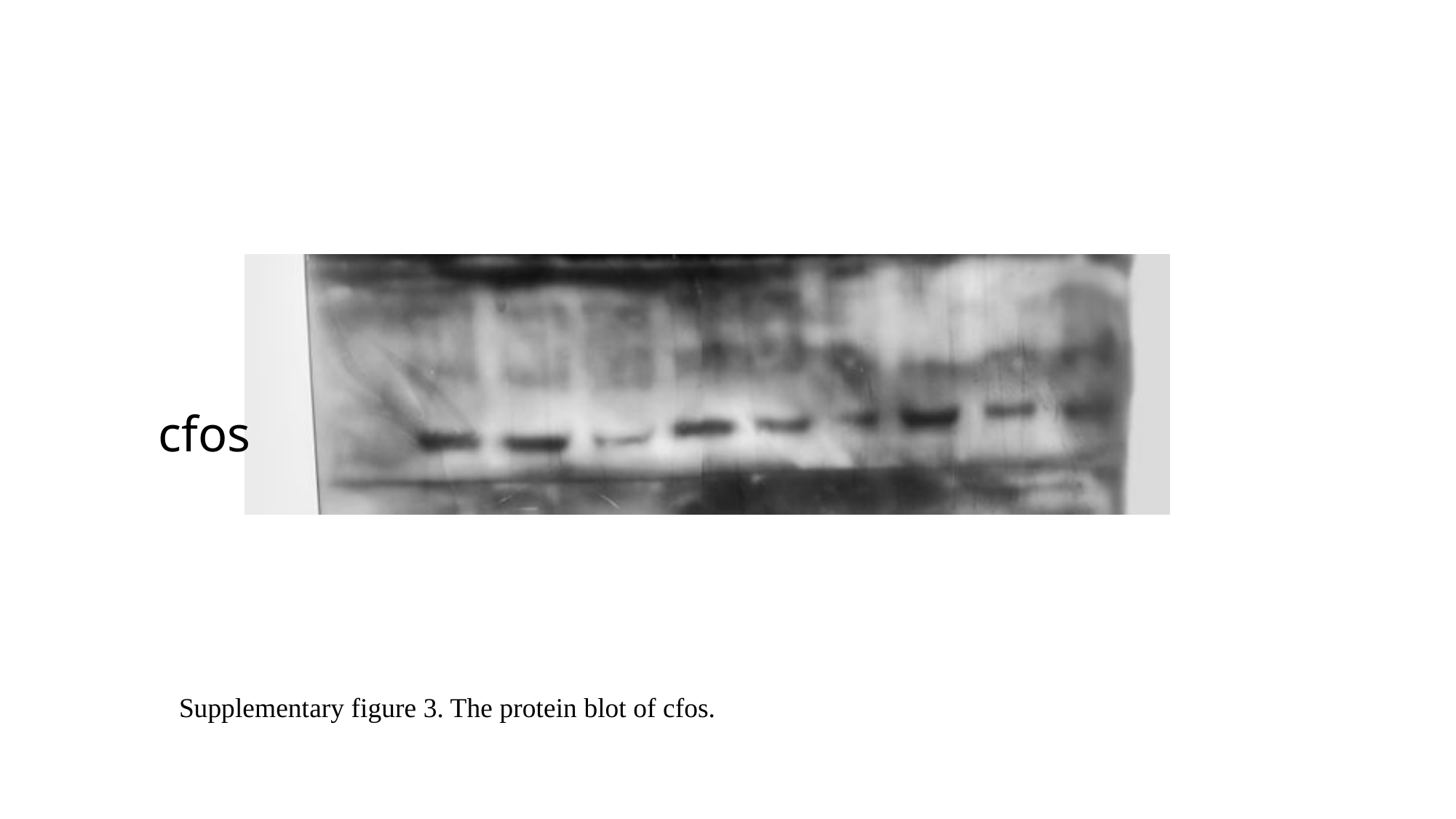

#
cfos
Supplementary figure 3. The protein blot of cfos.

## Slide 4
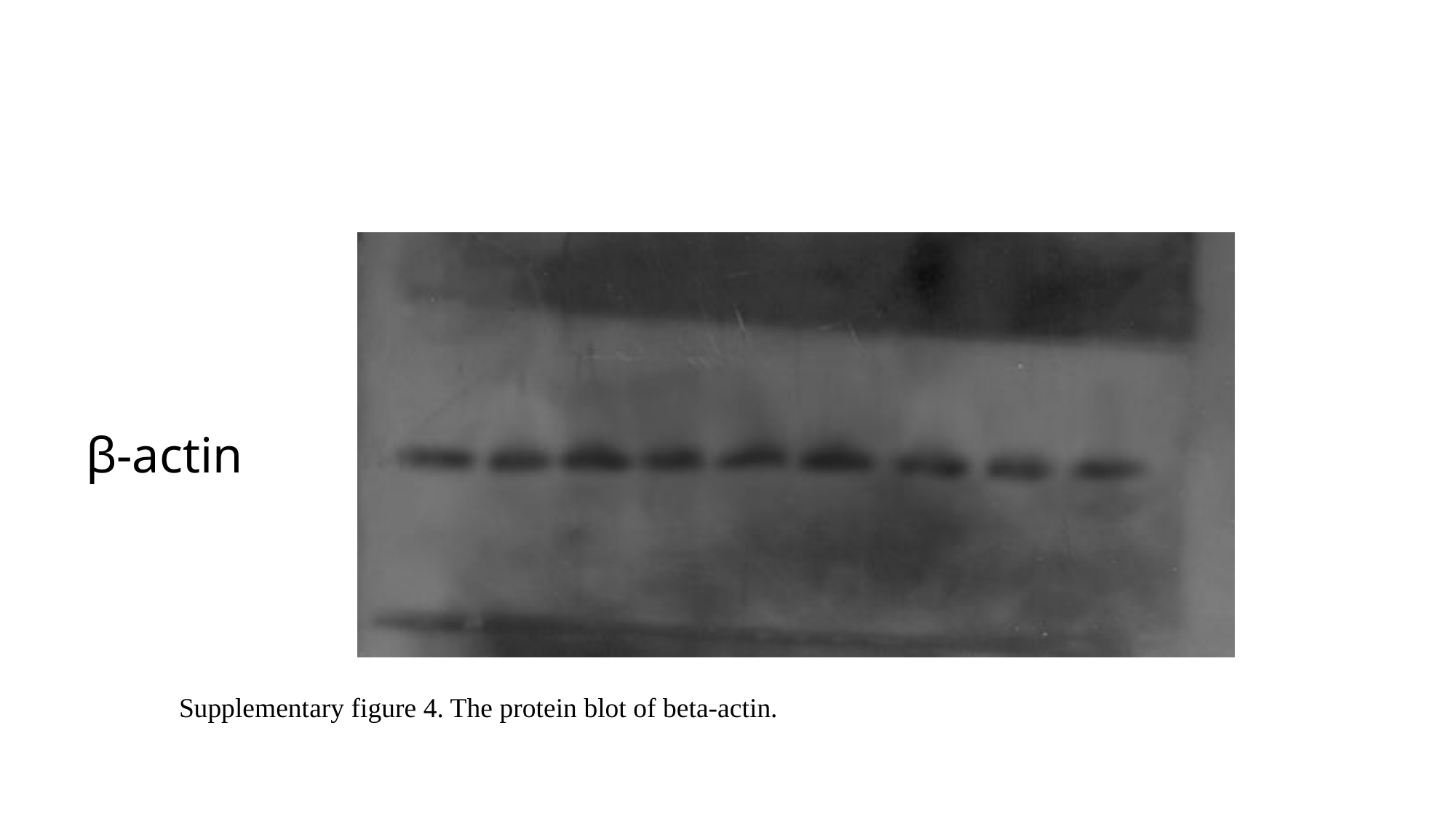

#
β-actin
Supplementary figure 4. The protein blot of beta-actin.
